# Supplementary material for: Ecology and Pathogenicity for Honey Bee Brood of Recently Described Paenibacillus melissococcoides and Comparison With Paenibacillus dendritiformis, Paenibacillus thiaminolyticus
Source: Environ Microbiol Rep. 2025 May 6;17(3):e70089. doi: 10.1111/1758-2229.70089 (PMC12056235; doi:10.1111/1758-2229.70089)

**Figure S1**: Number of colony forming units in suspensions of *Paenibacillus melissococcoides*, *Paenibacillus dendritiformis* and *Paenibacillus thiaminolyticus* immediately before, 1.5 and 4h after mixing with the diet. The number of spores in the suspensions was calculated from the percentage of spores evaluated by microscopy before mixing with the diet.


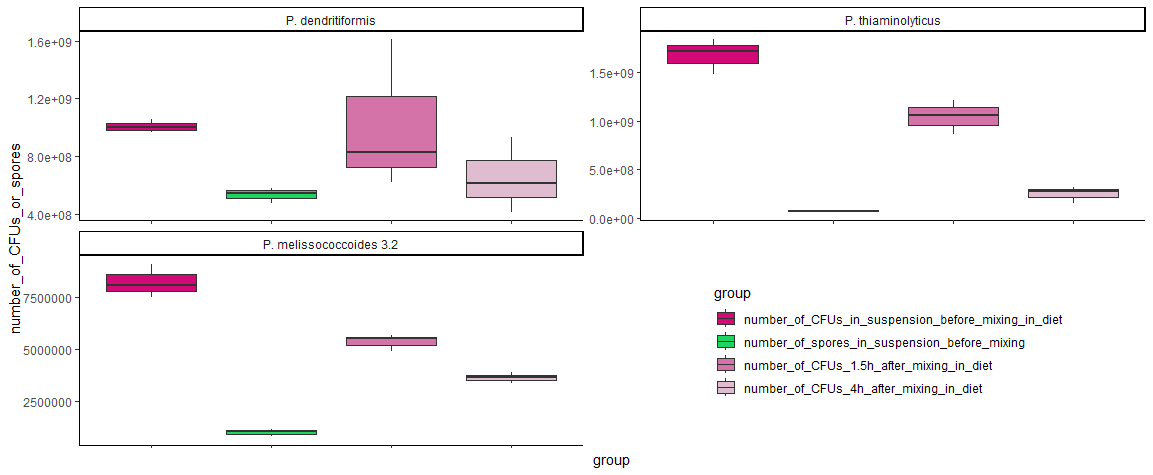

Supplement: Supplementary file 2 — Figure S1. Number of colony‐forming units in suspensions of Paenibacillus melissococcoides, Paenibacillus dendritiformis and Paenibacillus thiaminolyticus immediately before, 1.5 and 4 h after mixing with the diet. The number of spores in the suspensions was calculated from the percentage of spores evaluated by microscopy before mixing with the diet. [file EMI4-17-e70089-s002.docx]
